# The Informational Blackout chronic pain and fatigue as rational outputs of a brain operating on degraded biological signals

**DOI:** 10.3389/fpain.2026.1896306

**Published:** 2026-08-12

**Authors:** Carla Pandolfi-Cuadrado

**Affiliations:** Miguel Hernández University of Elche, Elche, Spain

**Keywords:** chronic pain, chronodisruption, circadian, endocannabinoid, fatigue, fibromyalgia, gut microbiota, Informational Blackout

## Abstract

Chronic pain and fatigue have resisted pharmacological resolution for two decades despite detailed mechanistic characterisation of nociceptive circuits. This perspective argues that the problem is not insufficient data but a mismatch between the level at which the dominant model operates and the level at which chronification is decided. Central sensitisation describes how nociceptive circuits amplify their gain. It does not, on its own, explain why that amplification becomes permanent, why it co-emerges systematically with fatigue and autonomic dysregulation, or why its epidemiology is stratified by sex in ways that differ qualitatively rather than quantitatively. The Informational Blackout framework proposes that chronic pain and fatigue are not malfunctions but appropriate responses from a self-maintaining system that has lost the biological infrastructure on which it was built to depend. The upstream driver is structural degradation of the signal architecture by the modern environment, mediated through SOCS3-driven multichannel transduction collapse and a convergent five-arm ionic setpoint failure that inscribes pain as a stable engram. The framework is offered as an upstream extension of central sensitisation; it grades its own evidence explicitly and generates falsifiable predictions that reorient therapeutic logic from nociceptive suppression toward signal architecture restoration.

## What the dominant model leaves unexplained?

Chronic pain persists even after tissue damage has healed. Fatigue deepens when the body rests. Neither symptom obeys the logic of central sensitisation on its own—a gap that has widened without a satisfying answer.

The data are well known: chronic pain affects one in five adults globally and is the leading single cause of years lived with disability (1). Yet, pharmacological response rates have not moved beyond 30%–40%, even after two decades of refinement (2, 3). This is not a failure of execution. It suggests that the dominant model, while valid at the circuit level, is incomplete at the level that governs whether a sensitised state stabilises or resolves.

Central sensitisation gave the field a real and well-supported foundation: a mechanistic account of how dorsal horn neurons increase their gain following sustained afferent drive, through N-methyl-D-aspartate (NMDA) receptor potentiation, microglial recruitment, and disinhibition (2, 4). As a description of synaptic events, it holds. What it does not provide, by itself, is an account of why those events become permanent—why the circuit stabilises in the sensitised state rather than returning to baseline once the initiating stimulus is removed.

Three structural, rather than incidental, limitations exist. First, the model treats the CNS as a circuit insulated from the organism’s metabolic, circadian, and immunological state; yet chronodisruption is a documented prognostic factor for pain chronification (5), and mitochondrial dysfunction in Schwann cells precipitates central changes that the sensitisation account does not predict (6). Second, it offers no principled reason why chronic pain clusters so reliably with fatigue, cognitive slowing, and autonomic dysregulation, as if these were incidental comorbidities rather than mechanistically linked outputs. Third, it does not ask what a sustained gain-increase state is for, or why a self-maintaining nervous system would stabilise it.

This framework begins with the last question, answering its two parts on its own terms. First, what the sustained gain increase is for: it is a protective output—the response a regulatory system produces when it must act on information it can no longer verify, restricting movement when structural integrity cannot be confirmed and restricting expenditure when energy states cannot be confirmed. Second, why it stabilises rather than resolving: because the mechanism that produces it is self-sustaining. As the following sections show, the collapse is held in place by molecular loops that reinforce one another and by the loss of the very signals that would be needed to revise the state, so the circuit cannot generate a different output even after the initiating injury is resolved. This is offered as an interpretive hypothesis: this perspective develops mechanistically the idea of a brain operating with near-optimal efficiency on the information available to it. The problem is not the response; it is the information, or the absence of it.

## The upstream driver: fragmentation of the biological signal architecture

The mammalian circadian system did not evolve to receive “a daily dose of light,” nor is it restored by the express-delivered melatonin promised for putting a screen down 60 min before bed—only to read under a bedside lamp whose blue light is dressed up as white. It evolved to extract structured information from a solar cycle that is chromatic, spectral, dynamic, and temporally progressive. Dedicated retinal circuits encode the blue-to-yellow transition of dawn as a discrete organising signal transmitted to the suprachiasmatic nucleus (SCN) (7). Near-infra-red (NIR) wavelengths, which are largely absent from indoor environments, support mitochondrial cytochrome c oxidase activity (8). The gut microbiota operate as an active zeitgeber, with microbiota-derived extracellular vesicles (MEVs) showing diurnal oscillation and modulating microglial transcriptional state on circadian timescales (9, 10).

These signals are not parallel inputs that happen to share a body. Together they form a structured zeitgeber network: a coordinated set of environmental and endogenous signals whose temporal coherence provides the brain with the biological reference for calibrating its internal state. This temporal structure does more than calibrate an internal reference: it schedules the organism’s circadian-gated restorative processes, such that its fragmentation removes not only the energy signal but also the temporal scaffold on which recovery, clearance, and repair are organised.

The modern environment does not simply attenuate this network; it fragments its structure. Artificial nocturnal light suppresses melatonin and eliminates the chromatic dawn signal, while indoor environments remove NIR. Misaligned feeding desynchronises peripheral metabolic clocks from SCN control, an effect compounded when nocturnal caloric intake activates suppressor of cytokine signalling 3 (SOCS3) in hypothalamic neurons through a pathway partially independent of circulating leptin levels and inflammatory cytokines (5, 11). Antibiotic use and dietary homogenisation flatten microbiota diurnal oscillations, converting a structured temporal signalling system into a constitutive neuroimmune load.

The result is not primarily a quantitative reduction in signal amplitude. Rather, it is a degradation of signal structure: inputs that are individually present but collectively incoherent, stripped of the temporal and spectral context that made them interpretable.

## The molecular gatekeeper: SOCS3 and multichannel transduction collapse

Leptin is not primarily a satiety hormone. Instead, it is the principal carrier of information about peripheral energy availability to the brain’s homeostatic architecture, operating through at least four functionally distinct channels: metabolic transcription via the Janus kinase/signal transducer and activator of transcription (JAK/STAT) pathway (12), tonic control over endocannabinoid synthesis, promotion of inhibitory synaptogenesis through STAT3–KLF4 (13), and transcriptional maintenance of the potassium–chloride co-transporter KCC2 in mature neurons (14).

SOCS3, induced by low-grade inflammation, hyperleptinemia, and circadian misalignment, blocks JAK2/STAT3, severing the molecular channel through which leptin reports bodily energy state. Its induction is rapid; its resolution is slow. Episodic chronodisruption produces cumulative JAK/STAT suppression that outlasts each desynchronisation episode, building toward a stable Informational Blackout (15).

Calling this “leptin resistance” frames a circuit-breaker as a dimmer switch. A more accurate description is a single molecular gatekeeper that interrupts four independent informational channels at once, with the qualification that the four channels are directly demonstrated in different cell types and disease contexts and their simultaneous collapse under a single upstream condition is an integrative proposal rather than a single established finding. A brain that cannot verify whether peripheral resources are genuinely depleted or whether the reporting infrastructure has failed responds rationally to the information it receives.

## Fatigue as a coordinated energy-conservation response

Fatigue is treated here not as a failure of energy production but as the appropriate output of a system that has lost the capacity to verify its own energy state. The same SOCS3 collapse that severs the leptin channels removes the brain’s principal report on peripheral resource availability, and a regulator that cannot confirm resources restricts expenditure. This extends the allostatic self-efficacy account of fatigue (16) by specifying the biological lesion—namely, the degradation of the afferent signals against which regulatory confidence is calibrated—rather than the failure of the regulatory action itself. The framework proposes that this conservation response is imposed at three convergent levels, explaining why fatigue co-emerges with pain rather than appearing as an independent symptom.

At the spinal level, these two symptoms share a descending substrate. The superficial neurokinin-1 receptor (NK1R)-expressing lamina I projection neurons that gate nociceptive transmission drive a spino-bulbo-spinal loop through the periaqueductal gray and the rostral ventromedial medulla (RVM), which returns descending serotonergic input to the dorsal horn (17). This descending drive is not uniformly analgesic. Its balance shifts toward facilitation in persistent pain, where RVM output maintains rather than initiates the sensitised state (18) through spinal 5-${\mathrm{HT}}_{3}$ receptors—the only excitatory ionotropic receptor of the serotonin family. Their activation drives a reciprocal neuron-glia cascade, fractalkine to microglia, IL-18 to astrocytes, IL-1β to neurons, and enhances NMDA activation, which feeds the microglial arm of the ionic setpoint collapse from above rather than from the periphery (19). The framework proposes that, as a testable extension rather than an established mechanism, under The Informational Blackout (Trademark) (53), the passive-defence response of the ventrolateral periaqueductal gray recruits this descending facilitation, so that a system built to quiet nociception instead raises the afferent cost of movement. This yields a falsifiable prediction: spinal 5-${\mathrm{HT}}_{3}$ blockade should attenuate fatigue-associated afferent amplification, not only nociceptive thresholds.

At the subcortical level, the same low-grade inflammation reduces striatal dopamine synthesis and release and steepens the discounting of effort, causing the brain to compute movement as not worth its energetic cost (20, 21). This is the motivational instantiation of the same conservation logic, supported by human and primate work, with its attribution to the leptin–SOCS3 axis as the inflammatory source remaining an inference of this framework. At the cellular level, circadian dysregulation of mitochondrial quality control shifts cells toward a protective hypometabolic state, forcing rest to preserve integrity (6). Cortisol amplitude flattening under chronodisruption is a further, covarying contributor that this perspective does not formalise; its integration is left as a necessary extension. This account of fatigue is distinct from, and testable against, established alternatives: it differs from cytokine-driven sickness behaviour (22) in persisting after active inflammation subsides, as its proximate driver is signal uninverifiability rather than cytokine levels; from sleep-deprivation fatigue in not resolving with sleep once the setpoint and descending facilitation have shifted; and from depression-associated fatigue in being predicted to track signal-coherence markers rather than mood. It treats mitochondrial dysfunction and autonomic dysregulation as convergent levels rather than origins, and predicts that ME/CFS and post-viral fatigue are maximal expressions of the same blackout, biomarker-stratified by signal coherence regardless of clinical label.

## Five-arm convergence on ionic setpoint collapse

The inhibitory polarity of GABAergic transmission in nociceptive circuits depends on the ratio of KCC2 to the sodium–potassium–chloride co-transporter NKCC1. Direct evidence from human post-mortem spinal cord tissue confirms that KCC2 expression is significantly reduced in donors with chronic pain compared to pain-free controls (23), establishing clinical relevance beyond animal models. At least five convergent molecular arms, all driven by the same upstream condition, shift this ratio toward the excitatory chloride configuration and lock it there. Because the arms differ in the extent to which each is demonstrated in adult nociceptive circuits, Table 1 grades the evidence for each explicitly, separating established findings from mechanistic inference and untested predictions.

**Table 1:** Evidence grading for the five convergent arms of ionic setpoint collapse.

Arm	Proposed mechanism	Tier	Load-bearing gap/prediction
Endpoint	Reduced KCC2 in human chronic-pain dorsal horn (23)	H	Causal directionality in humans untested
Arm 1	SOCS3 → STAT3 ↓ → KCC2 transcription ↓ (24, 25)	A/P	Direct STAT3–KCC2 coupling in adult dorsal horn (primary prediction)
Arm 2	Microglial BDNF → TrkB–Src → KCC2 phosphorylation (4, 26)	A	Human confirmation; female-route weighting
Arm 3	WNK1–SPAK ionic feedback ratchet (27)	A/I	Self-sustaining loop shown in vivo
Arm 4	Melatonin → BDNF/ERK1/2 → KCC2 restoration (28, 29)	A${}^{*}$/A	Direct melatonin–KCC2 in adult dorsal horn
Arm 5	ECS → CB1 → cAMP ↓ → KCC2 ↓ (30, 31)	A${}^{*}$/H	Functional arm in adult pain under leptin blackout

Evidence tiers: H, direct human evidence; A, animal evidence in adult nociceptive/dorsal horn circuits; A${}^{*}$, animal evidence in another circuit or developmental stage, extrapolated; I, mechanistic inference (links individually supported, integration in the pain context untested); P, untested falsifiable prediction.

**Arm 1, transcriptional suppression:** SOCS3-mediated STAT3 suppression is proposed to reduce KCC2 transcription in dorsal horn neurons. Leptin activates JAK2/STAT3 specifically in the spinal dorsal horn (24), and lentiviral SOCS3 overexpression in dorsal spinal glia reduces mechanical allodynia (25). The direct STAT3-to-KCC2 link in adult dorsal horn is the framework’s primary falsifiable prediction, awaiting direct in vivo confirmation.

**Arm 2, post-translational inactivation:** BK-channel hyperactivity in spinal microglia sustains constitutive brain-derived neurotrophic factor (BDNF) release, driving tropomyosin receptor kinase B (TrkB)–Src-mediated KCC2 phosphorylation at Tyr903 (26, 32). This arm predominates in male nociceptive circuits. The peripheral substrate sustaining microglial activation has a specific identity: Schwann cells under metabolic stress prioritise immediate lactate export to axons over myelin structural maintenance, an energetic sacrifice that generates tonic nociceptive input independent of overt mechanical injury (6), a sustained discharge pattern for which depolarising spontaneous fluctuations in peripheral sensory neurons provide the biophysical substrate (33).

**Arm 3, ionic feedback ratchet:** Elevated intracellular chloride from KCC2 downregulation activates the with-no-lysine kinase 1 (WNK1)–SPAK cascade, which further suppresses KCC2 while activating NKCC1 (27). This loop is proposed to sustain the collapse independently of upstream signals, once engaged—the mechanism that would make established chronic pain resistant to single-arm intervention.

**Arm 4, loss of endogenous restoration:** Melatonin upregulates KCC2 through BDNF/ERK1/2 signalling (28) and attenuates WNK–SPAK through nNOS modulation (34). Chronodisruption-induced melatonin deficiency removes this endogenous stabiliser. Recent evidence in nociceptive circuits shows that melatonin inhibits ERK/NF-κB in the spinal cord in neuropathic pain (29) and mitigates central sensitisation in fibromyalgia through MAPK modulation (35), narrowing but not closing the gap to a direct melatonin–KCC2 demonstration in the adult dorsal horn.

**Arm 5, endocannabinoid disinhibition:** SOCS3-mediated leptin blackout disinhibits synthesis in the endocannabinoid system (ECS). Elevated AEA and 2-AG are proposed to tonically activate type 1 cannabinoid (CB1) receptors, suppressing KCC2 through CB1-Gi-cAMP-PKA independently of the neuroinflammatory arms (30). CB1 receptors are preferentially localised in the superficial dorsal horn neuropil in both the adult rat and human spinal cord (31). Although the functional arm in adult pain remains a prediction, they are hypothesised to follow the same tonic-restraint logic by which leptin and melatonin normally hold their own targets in check. This tonic, KCC2-directed engagement is mechanistically distinct from the phasic presynaptic CB1 signalling that mediates cannabinoid analgesia. The framework predicts that sustained endocannabinoid elevation desensitises the latter, meaning that the excess proposed here presents downstream as a clinical endocannabinoid-deficiency signature (36).

Under these conditions, GABAergic polarity inversion enables long-term potentiation of nociceptive synapses that would normally remain subthreshold, and pain becomes inscribed as a stable engram maintained by the same ionic conditions that prevent its revision (3). The inversion also reinterprets classical gate control: under intact inhibition, large-fibre Aβ input helps close the gate on nociception (37); however, once KCC2 collapse inverts GABAergic polarity, disinhibition unmasks a polysynaptic Aβ input onto lamina I projection neurons, converting innocuous touch into a nociceptive drive—the circuit signature of tactile allodynia (38). The engram is not confined to the dorsal horn: sustained ascending input drives downstream sensitisation of the central amygdala and anterior cingulate cortex, consolidating the affective and cognitive dimensions of pain (39, 54), while circadian modulation of thalamic cholinergic gating provides a substrate for nocturnal amplification (40). If five independent arms sustain the collapse, no single-mechanism intervention can restore the inhibitory setpoint—a prediction quantitatively consistent with the 30%–40% response ceiling.

A second peripheral pathway potentiates the central collapse through gut serotonin. Chronodisruption-induced dysbiosis alters tryptophan hydroxylase 1 (TPH1) and serotonin transporter (SERT) expression in enterochromaffin cells, elevating peripheral 5-HT, which sensitises TRPA1 and TRPV1 channels on dorsal root ganglion neurons through 5-${\mathrm{HT}}_{3}$ and 5-${\mathrm{HT}}_{2A}$ receptors without requiring mechanical trauma (41, 42). The complete chain from sustained chronodisruption to downstream spinal sensitisation is a falsifiable prediction, which is not yet demonstrated in a single experimental model.

A single upstream switch ties these serotonergic and glutamatergic threads together. Tryptophan is the shared precursor of serotonin and the kynurenine pathway, and inflammation—by inducing indoleamine 2,3-dioxygenase—shunts it toward kynurenine (43). This lowers the substrate available for central serotonin synthesis, while the pathway’s two downstream metabolites act in opposition on the NMDA receptor and are cell-segregated: astrocytes produce the antagonist kynurenic acid, whereas activated microglia—the same cells that sustain the BDNF arm—produce quinolinic acid, an NMDA agonist and excitotoxin (43). Under microglial-dominant neuroinflammation, the balance tips toward quinolinic acid, supplying an endogenous NMDA agonist that feeds the pathological potentiation of the engram, in parallel with the GABAergic depolarisation that accompanies KCC2 collapse. The kynurenine pathway’s biology is established; its specific engagement in the dorsal horn under chronodisruption is a prediction of the framework, testable through the kynurenine-to-tryptophan ratio, which should rise with and predict both fatigue and nociceptive sensitisation.

## Sex differences are mechanistic signals, not epidemiological noise

The 3:1 to 9:1 female-to-male prevalence ratios in fibromyalgia, migraine, and temporomandibular disorders serve as mechanistic data (44). The framework predicts qualitatively distinct vulnerability profiles rather than quantitative gradations of the same mechanism; these are understood as probabilistic pathway biases modulated by hormonal status, reproductive stage, age, and prior injury, rather than fixed sex-specific mechanisms.

Two dimorphisms illustrate the pattern. Women operate at a higher basal leptin level through oestrogen-driven secretion (45), placing them chronically closer to the SOCS3 induction threshold, and they carry a higher basal melatonin amplitude (46), whose loss under chronodisruption results in a proportionally larger fall in the SOCS3 suppressor and KCC2 stabiliser. The same dimorphisms that confer resilience under optimal conditions produce disproportionate fragility under signal degradation.

The dominant route to collapse also appears to differ. In males, the microglial BK-BDNF-TrkB arm tends to predominate, and P2X4 receptor (P2X4R) blockade substantially reduces neuropathic pain behaviour in male rodents (32, 47); in females, the sensitised state weighs more heavily toward the peripheral bioenergetic arm, involving Schwann cell dysfunction, TRP channel miscalibration, and retrograde TrkB signalling (48). Convergence on KCC2 dysregulation as the final common mechanism is preserved; the route is not. A literature built predominantly on male subjects has characterised one arm while underdescribing the other, producing evidence more applicable to male pathophysiology than to the population bearing the greatest burden.

## Therapeutic reorientation as a research agenda

Suppressing the output of a system operating on degraded inputs does not restore those inputs. The proposed target is the signal architecture upstream, and the proposals below are presented as testable hypotheses rather than clinical recommendations.

A multimodal protocol that addresses at least three of the five arms simultaneously is predicted to outperform any single-mechanism approach. Melatonin chronotherapy at physiological doses, timed to the individual’s circadian phase, is proposed to address Arms 1–4 through its actions on SOCS3 suppression, BDNF/ERK1/2-mediated KCC2 upregulation, and WNK–SPAK modulation (28, 34, 49). Transcutaneous auricular vagus nerve stimulation is proposed to address Arms 2–4 through melatonin amplification (50) and cholinergic anti-inflammatory signalling. Time-restricted feeding aligned with the active circadian phase reduces SOCS3 induction and restores peripheral clock synchrony (5, 11). Morning chromatic light exposure restores the amplitude of the cortisol awakening response and the melatonin phase (7, 51).

Concretely, the framework specifies a testable design: a randomised controlled trial comparing the multimodal protocol against an active control; this trial includes circadian phase assessment at entry, salivary endocannabinoid profile and cortisol awakening response amplitude as primary mechanistic endpoints, nociceptive thresholds and fatigue as secondary endpoints, sex-stratified analysis at the nodal level, and sufficient power to detect the ordering the framework predicts. That prediction is the discriminating one: under upstream multimodal intervention, salivary endocannabinoid profiles and cortisol awakening response amplitude should normalise *before* nociceptive thresholds and fatigue improve. Central sensitisation models predict the reverse ordering, and allostatic-load models predict simultaneous change; this temporal precedence is therefore the critical test of causal directionality.

## The disease is the silence

Chronic pain and fatigue are not errors; they are the responses of a self-maintaining nervous system operating correctly in the absence of reliable data, having lost the interoceptive coordination it depends on. The central, testable claim that follows is narrow and specific: if the framework is correct, restoration of biological signal coherence should temporally precede and predict improvement in pain and fatigue, and a failure of that ordering would require a revision of the model.

Whether the same chronification architecture generalises beyond chronic pain to other conditions that share chronodisruption, metabolic, and neuroinflammatory features is an empirical question pursued in a separate mechanistic taxonomy (52) and left outside the scope of this pain-focused perspective. What the framework offers within that scope is an upstream cause for a state that circuit-level models describe but do not explain and a reason a self-maintaining organism would hold it in place.

The disease is the silence where the signal used to be—and that silence, this framework proposes, has a molecular address.

## Data Availability

The original contributions presented in the study are included in the article/Supplementary Material; further inquiries can be directed to the corresponding author/s.

## Author’s note

This perspective is based on the working paper *The Informational Blackout: A Unified Framework for Chronic Pain and Fatigue Through Loss of Foundational Biological Coordination* (Pandolfi Cuadrado, 2026), deposited in open access on OSF. DOI: 10.17605/OSF.IO/HJWC7.
